# Naming racism as a root cause of inequities in palliative care research: a scoping review

**DOI:** 10.1186/s12904-024-01465-9

**Published:** 2024-06-10

**Authors:** Kavita Algu, Joshua Wales, Michael Anderson, Mariam Omilabu, Thandi Briggs, Allison M. Kurahashi

**Affiliations:** 1 Temmy Latner Centre for Palliative Care, 60 Murray Street, 4th Floor, Box 13, Toronto, ON M5T3L9 Canada; 2 Waakebiness-Bryce Institute for Indigenous Health, Dalla Lana School of Public Health, University of Toronto, 155 College Street, 6th floor, Toronto, ON M5T 3M7 Canada; 3 Home and Community Care Support Services Toronto Central, 250 Dundas St. W, Toronto, ON M5T 2Z5 Canada

**Keywords:** Palliative care, Health equity, Race and ethnicity, Racism

## Abstract

**Background:**

Racial and ethnic inequities in palliative care are well-established. The way researchers design and interpret studies investigating race- and ethnicity-based disparities has future implications on the interventions aimed to reduce these inequities. If racism is not discussed when contextualizing findings, it is less likely to be addressed and inequities will persist.

**Objective:**

To summarize the characteristics of 12 years of academic literature that investigates race- or ethnicity-based disparities in palliative care access, outcomes and experiences, and determine the extent to which racism is discussed when interpreting findings.

**Methods:**

Following Arksey & O’Malley’s methodology for scoping reviews, we searched bibliographic databases for primary, peer reviewed studies globally, in all languages, that collected race or ethnicity variables in a palliative care context (January 1, 2011 to October 17, 2023). We recorded study characteristics and categorized citations based on their research focus—whether race or ethnicity were examined as a major focus (analyzed as a primary independent variable or population of interest) or minor focus (analyzed as a secondary variable) of the research purpose, and the interpretation of findings—whether authors directly or indirectly discussed racism when contextualizing the study results.

**Results:**

We identified 3000 citations and included 181 in our review. Of these, most were from the United States (88.95%) and examined race or ethnicity as a major focus (71.27%). When interpreting findings, authors directly named racism in 7.18% of publications. They were more likely to use words closely associated with racism (20.44%) or describe systemic or individual factors (41.44%). Racism was directly named in 33.33% of articles published since 2021 versus 3.92% in the 10 years prior, suggesting it is becoming more common.

**Conclusion:**

While the focus on race and ethnicity in palliative care research is increasing, there is room for improvement when acknowledging systemic factors – including racism – during data analysis. Researchers must be purposeful when investigating race and ethnicity, and identify how racism shapes palliative care access, outcomes and experiences of racially and ethnically minoritized patients.

**Supplementary Information:**

The online version contains supplementary material available at 10.1186/s12904-024-01465-9.

## Introduction

Significant racial and ethnic disparities in palliative care access and outcomes [1–3]. For example, racialized patients have been found to have decreased access to culturally appropriate palliative care [4–6], and unequal access to symptom management medications resulting in worse pain management [7–9]. Research from the United States (US) shows that when accessed, racialized groups are more likely to disenroll from hospice [10–12]. Researchers are being encouraged to include analyses of racism when investigating and explaining disparities [13] as an acknowledgement that health inequities experienced by racialized and ethnically minoritized people are “rooted in racism shaped by the legacies of slavery, indentured servitude, colonialism, imperialism, war, ultra-nationalism, ethnic absolutism, xenophobia and hate speech” [14]. Some palliative care journals now require researchers who include race as a variable to explicitly “address structural racism, calling it out by name, and identifying the form it takes” when interpreting findings [15].

The manner in which researchers interpret their findings holds the power to shape future interventions that reduce and eliminate disparities: the root causes posited by researchers to explain health disparities or their determinants are more likely to be investigated in subsequent research studies. In turn, these inform the evidence-based interventions and policy changes designed to reduce or eliminate disparities [16, 17]. To make substantive contributions to reducing disparities in palliative care, researchers investigating race and ethnicity have a responsibility to name and discuss the role of racism when interpreting their findings. If racism is not named as a root cause, it is less likely to be addressed, and inequities will persist [14]. Despite the recognized value of naming racism, little is known about how often, if at all, systemic and interpersonal racism are named in the contextualization of findings in palliative care disparities research.

Our study aims to summarize the characteristics of 12 years of academic literature that investigates race- or ethnicity-based disparities in palliative care access, outcomes and experiences, and determine the extent to which racism is discussed when interpreting findings.

### Terminology

To position this research, the concepts of race, ethnicity, and racism need to be defined and contextualized (Table 1). Race is a social construct [14] created to facilitate the exploitation and abuse of people experiencing colonization by European imperialist nations [18]. As a social construct, race has no bearing on biological differences and is not due to genetic variations [19]; therefore, health disparities do not result from race itself, but rather from the experiences of racism at multiple societal levels [20]. The exact definitions, meanings, and categories pertaining to race can change depending on the larger context (e.g., the same person may be identified as different races in different countries). Some countries do not widely discuss race at all, but discrimination based on colourism or ethnic group may occur [21–23]. The present-day concepts of race and racism used in this review are drawn from the definitions describing power relationships rooted in colonial legacies and practices which may not be ubiquitous or consistently conceptualized across all countries. Ethnicity is a grouping of people based on shared history, territory, or culture, and does not specifically include race. Because many citations included in this review erroneously used these terms interchangeably, we often refer to them together, acknowledging the limitations of this approach.

Racism broadly refers to conscious or unconscious oppression, inferior treatment, or lower status assigned to a racialized or ethnically minoritized individual or group [24]. Systemic racism, structural racism, and institutional racism are related but distinct terms that refer to racism embedded within the fabric and structures of society, policies, and institutions [24, 25]. These manifestations of racism differ from interpersonal racism, which occurs between two or more individuals [26].

**Table 1:** Important definitions of race, ethnicity and racism

Term	Definition and usage
Race	A socially constructed grouping together of people based on physical features, initially created to marginalize and exploit groups of people [27]
Ethnicity	Grouping based on factors such as shared ancestry and culture [28]
Racism	Oppression or inferior treatment or status assigned to racialized individuals or groups – whether intentional or unintentional/unconscious [24]
Systemic racism	Whole societal systems that perpetuate racial injustices including “political, legal, economic, healthcare, school and criminal justice systems”. These systems may not always be visible, particularly to those who are not negatively impacted by them. Systemic racism includes structural and institutional racism and the word systemic racism is often used to refer to all three of these concepts together [24]
Structural racism	The specific structures that hold up systemic racism, for example, laws, policies, and institutional practices [24]
Institutional racism	Racism within a specific institution, or within institutional systems [24]
Interpersonal racism	Racism within an interaction between two or more individuals [26]

## Methods

### Research design

We followed Arksey & O’Malley’s methodology for scoping reviews [29]. Compared to systematic reviews, which critically appraise and synthesize evidence to answer a single or precise research question, scoping reviews concentrate on an overall body of literature and can be used to examine how research is conducted on a certain topic [30]. We felt that a scoping review methodology best aligned with our study aim to summarize the investigation and interpretation of research that contributes to detecting and understanding race- and ethnicity- based disparities in palliative care. We describe our review following the PRISMA Extension for Scoping Reviews (PRISMA-ScR) reporting [31].

### Procedure

#### Identifying relevant literature

In consultation with an Information Specialist, we designed a search strategy to be applied across multiple bibliographic databases for publications between January 1, 2011 and October 17, 2023. We searched bibliographic databases focused on medicine and healthcare (CINAHL, EMBASE, MEDLINE, PubMed, Cochrane) for terms broadly centered around palliative care, race and ethnicity. We selected broad terms to increase the potential of identifying articles that may refer to these concepts using different terms. A comprehensive list of databases and search terms are in Additional file 1. We ran the initial search in July, 2021, and two subsequent searches in August 2022 and October 2023. While we did not include reviews in our analysis, we did hand–search the reference list for review studies that passed title and abstract screening.

#### Inclusion and exclusion criteria

We conducted a broad, comprehensive search, not limiting inclusion to any disease, country of origin, or language of publication. We structured our inclusion and exclusion criteria (Table 2) to focus on peer-reviewed studies that contributed to detecting or understanding race- or ethnicity-based disparities related to palliative care access, outcomes or experiences. During the screening phases, we evaluated each citation and included studies that (1) took place within a palliative care context (i.e., care was delivered in a palliative or hospice program, or by a palliative care or hospice provider); (2) collected and analyzed race or ethnicity as an independent variable, or used a race or ethnicity to define a study population or cohort; and (3) was peer-reviewed and evaluated primary data. Because our review focused on articles that describe and explain disparities, we excluded articles that evaluated interventions to reduce disparities as these primarily focus on solving a problem, not describing it.

**Table 2:** Inclusion and exclusion criteria

Category	Inclusion Criteria	Exclusion Criteria
Palliative Care	• **Program**: Care was or would be provided in a hospice or palliative care program, *or* • **Provider**: Care was or would be delivered by a palliative care or hospice provider, *and* • Participants had directly received, delivered or referred to palliative care or hospice care	• The following were not considered palliative care unless provided within a palliative/hospice setting or by a palliative/hospice provider o End-of-life care (e.g., traumatic injuries) o Advance care planning/goals of care discussions o Treatments such as palliative radiotherapy, chemotherapy, surgery, etc. o Medical Assistance in Dying or Physician Assisted Death o Do not resuscitate/code status studies • Participants had no direct exposure to palliative care (e.g., asking general population about their awareness of palliative care, feelings about dying, etc. )
Race and Ethnicity	• Collected and analyzed “race” or “ethnicity” as an independent variable, or used a race or ethnicity to define a study population or cohort • Articles focused on a specific racial or ethnic group that would be considered minoritized in the country the study was performed in	• Race and ethnicity were only presented as participant demographic variables (e.g., as in a Table 1) • Studies focused only on other concepts sometimes associated with race/ethnicity such as: o Language o Immigration o Nationality o Culture o Spirituality
Study type	• Peer-reviewed articles reported following the IMRaD format • Non-interventional studies focused on detecting or understanding racial or ethnic disparities • Studies using primary data^a^	• Abstracts and conference proceedings • Interventional studies focused on reducing racial or ethnic disparities • Studies examining patient preferences but without evaluating congruence between desired and actual care • Review articles^a^

^a^ These criteria were applied during full text screen, only

#### Title and abstract screen

Two of three reviewers (KA, AK, JW) independently screened each title and abstract to assess eligibility. Differences were resolved through discussion, or assessment by a third reviewer. When it was unclear whether an article met criteria, it was included in the full text screening. If an article title appeared relevant but an abstract was not available, we included it in the full review. All articles in other languages had abstracts available in English; none of these articles were included in the full text screen.

#### Full text screen

The full text of eligible citations was screened using the same criteria as above and review articles were excluded. We hand–screened the reference lists from review articles that passed the title and abstract screen phase to identify any additional eligible citations.

#### Charting the data

During data abstraction, two independent reviewers (KA and AK) both recorded information for the first 45 articles, ensuring a common understanding. One reviewer (KA or AK) abstracted data for the remaining citations, and each reviewed the other’s data at the end of the abstraction process. Using a standardized form (Additional file 2) for each citation we documented: first author, title, publication year, location, study purpose, study outcomes, races or ethnicities collected, and whose race or ethnicity was collected.

We initially captured nine categories for race and ethnicity including ‘other: please specify’. Following a review of the free–text responses provided in the ‘other’ category, we created five additional categories and reclassified ‘other’ responses accordingly. These include Asian/Pacific Islander, Turtle Island (North American) Indigenous, Aboriginal Australian, Māori, and Mixed race. The new ‘other’ category captured instances when researchers included an ‘other’ option, or when they included an exclusionary group (e.g. “non-Hispanic”, “non-White”, “none of the above”, etc.).

The study team observed a great degree of heterogeneity among the included articles. In line with scoping review methodology, which affords an iterative approach to data analysis with increasing familiarity of the literature [32, 33], we further classified studies based on whether the examination of race or ethnicity was the major or minor focus of the research purpose (research focus), and whether authors directly or indirectly discuss racism when interpreting their findings (interpretation of findings). The operationalization of these variables is described below and in Table 3.

##### Research focus

We reviewed the study purpose and classified studies as having a ‘major focus’ if race or ethnicity were named in the study purpose, and the study was designed to (a) evaluate associations between palliative care access, outcomes or experiences and patient or provider race or ethnicity, or (b) investigate the experiences of patients or providers representing a specific racial or ethnic population. We classified studies as ‘minor focus’ if race or ethnicity were not named in the study purpose, and the study was designed to evaluate associations between palliative care access, outcomes or experiences and a variable other than race or ethnicity, but race or ethnicity were analyzed as one of several secondary characteristics or variables.

##### Interpretation of finding

We reviewed the discussion section of each article for mention of racism according to four binary variables ranging from direct to indirect: (1) named racism (yes/no), (2) used a keyword associated with racism (yes/no), (3) described systemic or individual provider factors (yes/no), or (4) none (yes/no). To determine if authors explicitly named racism or used a keyword, we conducted a text search for the terms racis*, bias, discriminat*, systemic, prejudic*, or stereotype. We reviewed the use of each term in context to verify it pertained to disparities. These keywords were selected based on concepts presented in previously published author guidelines and recommendations for palliative care research that includes the examination of race, as well as closely related synonyms [15, 20]. Next, we looked for text describing systemic or individual provider factors that may impact racially or ethnically minoritized individuals. For example, describing differential access to healthcare resources in neighborhoods serving predominantly racially minoritized patients (including hospital funding or staffing); opiate prescribing patterns by physicians; comments on cultural sensitivity; historical trauma resulting in patient mistrust; institutional policies, practices and regulations; or social determinants of health. These factors impact racially or ethnically minoritized individuals and are often associated with systemic racism (including structural and institutional racism) and interpersonal/individual racism (including prejudice or bias). Finally, citations that did not use any of these were marked as yes for ‘none’.

**Table 3:** Operational definitions of variables

Research focus	Were race or ethnicity a major or minor focus of the research purpose? **1)** **Major focus: **The study was designed to evaluate associations between palliative care access, outcomes or experiences and patient/provider race or ethnicity **or** to investigate the experiences of patients/providers representing specific racial or ethnic populations. (i.e., race or ethnicity were treated as a primary independent variable, used for cohort construction or guided purposeful sampling of participants) **2)** **Minor focus: **Race or ethnicity were not named in the study purpose and were included in secondary analysis
Interpretation of findings	Did authors directly or indirectly discuss racism when interpreting their findings? 1) **Named racism: **Authors used the word racis* when interpreting their findings 2) **Used a keyword** associated with racism**: **Authors used one of the terms bias, discriminat*, systemic, prejudic*, or stereotype when interpreting their findings 3) **Described systemic or individual provider factors:** Authors described systemic or individual provider factors when interpreting their findings 4) **None**: The authors did not directly or indirectly discuss any systemic, structural, institutional or interpersonal/individual factors

### Data analysis

We summarized study characteristics as well as the distribution of articles by research focus and interpretation of findings as overall counts and percentages. We evaluated changes in research focus and interpretation of findings over time by counting the number of citations in each category year by year. Finally, we explored the relationship between research focus and interpretation of findings by visualizing the flow of citations from research focus across variables, from indirect to direct references to racism.

## Results

**Fig. 1:**
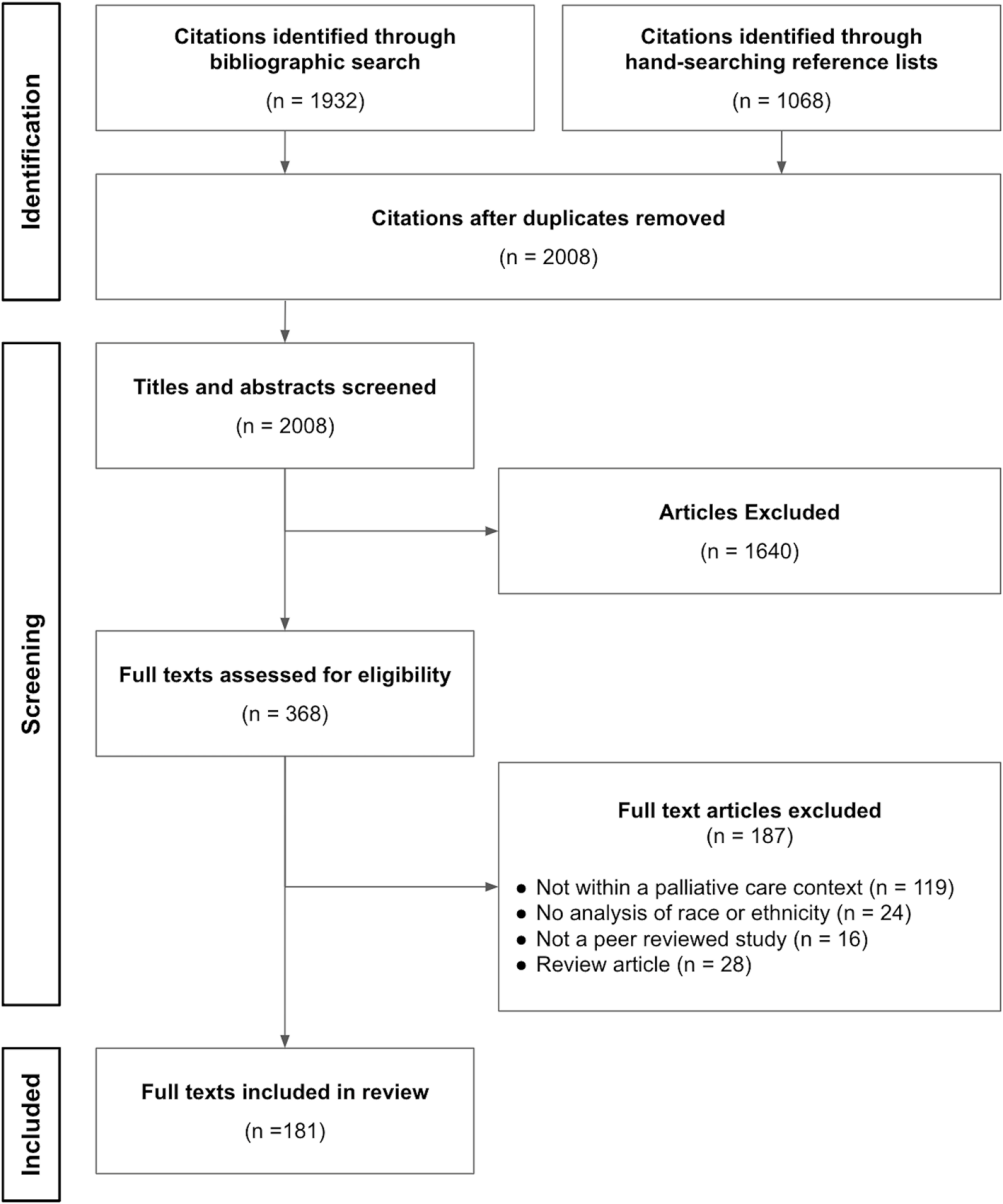
PRISMA flow diagram

We identified 3000 articles (See PRISMA diagram in Fig. 1). Following title and abstract screening, 368 articles remained and were included in the full text screening. The final review includes 181 articles, whose characteristics are detailed in Table 4.

**Table 4:** Study Characteristics

Study Information					Research Focus	Interpretation of Findings		
**First Author (Year)**	**Study Country**	**Study Design**	**Races/Ethnicities Represented**	**Race/Ethnicity collected for**	**Race/ethnicity was a major focus of the research purpose** ^**a**^	**The authors named racism in the discussion** ^**a**^	**The authors used** **a** **keyword** ^**b**^ **in the discussion** ^**a**^	**The authors described systemic or individual provider factors** ^**a**^
**Loggers (2013)** [34]	**US**	**Quantitative**	**Latinx/Hispanic, White**	**Patient/Caregiver**	**⚫**			⚫
**Yang (2020)** [35]	**US**	**Quantitative**	**Black, Latinx/Hispanic, White, Asian/Pacific Islander**	**Patient/Caregiver**	**⚫**		⚫	⚫
**Wen (2019)** [36]	**US**	**Quantitative**	**Black, Latinx/Hispanic, Asian | Multiple Groups, White**	**Patient/Caregiver**	**⚫**			⚫
**Rice (2021)** [37]	**US**	**Quantitative**	**White, Other**	**Patient/Caregiver**				
**Lillemoe (2020)** [38]	**US**	**Quantitative**	**Black, LatinX/Hispanic, Asian | Multiple Groups, White, Unknown**	**Patient/Caregiver**			⚫	
**Wang (2019)** [11]	**US**	**Quantitative**	**Black, Latinx/Hispanic, Asian | Multiple Groups, White, Unknown**	**Patient/Caregiver**	**⚫**			
**Unroe (2012)** [10]	**US**	**Quantitative**	**White, Other**	**Patient/Caregiver**	**⚫**			⚫
**Chuang (2017)** [39]	**US**	**Quantitative**	**Black, White, Mixed, Other**	**Patient/Caregiver**	**⚫**		⚫	⚫
**Shin (2015)** [8]	**US**	**Quantitative**	**Black, Latinx/Hispanic, Asian | Multiple Groups, White, Other**	**Patient/Caregiver**				
**Sharma (2011)** [40]	**US**	**Quantitative**	**Black, White**	**Patient/Caregiver**				⚫
**Taylor (2019)** [41]	**US**	**Quantitative**	**Black, Latinx/Hispanic, White, Other**	**Patient/Caregiver**	**⚫**			
**Cea (2016)** [9]	**US**	**Quantitative**	**Black, Latinx/Hispanic, White, Other**	**Patient/Caregiver**			⚫	⚫
**Colon (2012)** [42]	**US**	**Qualitative**	**Latinx/Hispanic**	**Patient/Caregiver**	**⚫**			⚫
**Ernst (2021)** [43]	**US**	**Quantitative**	**East Asian, White**	**Patient/Caregiver**	**⚫**			
**Subramaniam (2021)** [44]	**US**	**Quantitative**	**Black, White, Other**	**Patient/Caregiver**	**⚫**			
**Miesfeldt (2012)** [45]	**US**	**Quantitative**	**Black, Other**	**Patient/Caregiver**	**⚫**			⚫
**Wang (2016)** [46]	**US**	**Quantitative**	**Black, Latinx/Hispanic, White, Other**	**Patient/Caregiver**				
**Chen (2020)** [47]	**US**	**Quantitative**	**Black, Asian | Multiple Groups, White, Other, Unknown**	**Patient/Caregiver**			⚫	⚫
**Rosenfeld (2018)** [48]	**US**	**Quantitative**	**Black, Latinx/Hispanic, Asian | Multiple Groups, White, Other**	**Patient/Caregiver**				
**Cruz–Flores (2019)** [49]	**US**	**Quantitative**	**Black, Latinx/Hispanic, White, Asian/Pacific Islander, Other**	**Patient/Caregiver**	**⚫**			
**Johnson (2013)** [50]	**US**	**Quantitative**	**Black, White**	**Patient/Caregiver**	**⚫**			
**Rhodes (2012)** [51]	**US**	**Quantitative**	**Black**	**Patient/Caregiver**	**⚫**			⚫
**Ache (2011)** [52]	**US**	**Quantitative**	**Black, White**	**Provider**	**⚫**			⚫
**Prince (2019)** [53]	**Canada**	**Qualitative**	**North American Indigenous**	**Patient/Caregiver, Provider**	**⚫**			⚫
**Slater (2015)** [54]	**New Zealand**	**Qualitative**	**Māori**	**Patient/Caregiver, Provider**	**⚫**			⚫
**Rhodes (2013)** [55]	**US**	**Quantitative**	**Black, Latinx/Hispanic, Asian | Multiple Groups, White, Other**	**Patient/Caregiver**	**⚫**			
**Hardy (2012)** [56]	**US**	**Quantitative**	**Black, Latinx/Hispanic, Asian | Multiple Groups, White, Asian/Pacific Islander**	**Patient/Caregiver**	**⚫**		⚫	⚫
**Kirkendall (2015)** [57]	**US**	**Quantitative**	**Latinx/Hispanic**	**Patient/Caregiver**	**⚫**			
**Karanth (2018)** [58]	**US**	**Quantitative**	**Black, Latinx/Hispanic, White, Other**	**Patient/Caregiver**	**⚫**		⚫	⚫
**Hughes (2020)** [59]	**US**	**Qualitative**	**Other**	**Patient/Caregiver**	**⚫**			⚫
**Colon (2015)** [60]	**US**	**Quantitative**	**Black, Latinx/Hispanic, Asian | Multiple Groups, White**	**Patient/Caregiver**	**⚫**			
**Beltran (2018)** [61]	**US**	**Quantitative**	**Latinx/Hispanic, White**	**Patient/Caregiver**	**⚫**			⚫
**Bell (2011)** [62]	**US**	**Quantitative**	**Asian | Multiple Groups, White, Asian/Pacific Islander, Other**	**Patient/Caregiver**	**⚫**			
**Khan (2022)** [63]	**US**	**Quantitative**	**Black, Latinx/Hispanic, White, Asian/Pacific Islander, North American Indigenous, Other**	**Patient/Caregiver**	**⚫**		⚫	
**Karikari–Martin (2016)** [64]	**US**	**Quantitative**	**Black, White**	**Patient/Caregiver**	**⚫**			⚫
**Dillon (2016)** [65]	**US**	**Qualitative**	**Black**	**Patient/Caregiver**	**⚫**		⚫	⚫
**Rubens (2019)** [66]	**US**	**Quantitative**	**Black, Latinx/Hispanic, Asian | Multiple Groups, White, Other, Asian/Pacific Islander**	**Patient/Caregiver**				
**Enguidanos (2013)** [67]	**US**	**Quantitative**	**Black, Latinx/Hispanic, White, Other**	**Patient/Caregiver**	**⚫**			
**Faigle (2017)** [68]	**US**	**Quantitative**	**White, Other**	**Patient/Caregiver**	**⚫**			⚫
**Kathpalia (2016)** [69]	**US**	**Quantitative**	**Black, Latinx/Hispanic, Asian | Multiple Groups, White, Other**	**Patient/Caregiver**				
**Paredes (2021)** [70]	**US**	**Quantitative**	**White, Other**	**Patient/Caregiver**	**⚫**			
**Laguna (2014)** [71]	**US**	**Quantitative**	**Black, Latinx/Hispanic, Asian | Multiple Groups, White, North American Indigenous, Other**	**Patient/Caregiver**	**⚫**			
**Russell (2017)** [12]	**US**	**Quantitative**	**Black, Latinx/Hispanic, Asian | Multiple Groups, White, Other**	**Patient/Caregiver**				
**Maddalena (2013)** [72]	**Canada**	**Qualitative**	**Black**	**Patient/Caregiver**	**⚫**	⚫	⚫	⚫
**Haines (2018)** [73]	**US**	**Quantitative**	**Black, Latinx/Hispanic, Asian | Multiple Groups, White, North American Indigenous**	**Patient/Caregiver**	**⚫**			
**Dembinsky (2014)** [74]	**Australia**	**Qualitative**	**Aboriginal Australian**	**Provider**	**⚫**			⚫
**Woods (2020)** [75]	**Australia**	**Quantitative**	**Aboriginal Australian**	**Patient/Caregiver**	**⚫**			
**Shiovitz (2015)** [76]	**US**	**Quantitative**	**White, North American Indigenous**	**Patient/Caregiver**	**⚫**			
**Neiman (2019)** [77]	**US**	**Qualitative**	**Asian | Multiple Groups**	**Patient/Caregiver**	**⚫**			⚫
**Reyes–Gibby (2012)** [78]	**US**	**Quantitative**	**Black, Latinx/Hispanic, White**	**Patient/Caregiver**	**⚫**			⚫
**Woods (2021)** [79]	**Australia**	**Quantitative**	**Aboriginal Australian**	**Provider**	**⚫**			
**Gerlach (2021)** [7]	**US**	**Quantitative**	**Latinx/Hispanic, White, Other**	**Patient/Caregiver**			⚫	
**Austin (2019)** [80]	**US**	**Quantitative**	**Black, White**	**Patient/Caregiver**	**⚫**			⚫
**Teno (2016)** [81]	**US**	**Quantitative**	**Black, Latinx/Hispanic, Asian | Multiple Groups, White, Other**	**Patient/Caregiver**				
**Rhodes (2015)** [82]	**US**	**Qualitative**	**Black**	**Patient/Caregiver**	**⚫**			⚫
**Price (2017)** [83]	**US**	**Quantitative**	**Black, Latinx/Hispanic, White, Other**	**Patient/Caregiver**	**⚫**			
**Nadimi (2011)** [84]	**Australia**	**Quantitative**	**Aboriginal Australian, White**	**Patient/Caregiver**	**⚫**			
**Ornstein (2020)** [85]	**US**	**Quantitative**	**Black, White**	**Patient/Caregiver**				
**Thongprayoon (2021)** [86]	**US**	**Quantitative**	**Black, Latinx/Hispanic, White, Other**	**Patient/Caregiver**				
**Dillon (2015)** [87]	**US**	**Qualitative**	**Black**	**Patient/Caregiver**	**⚫**			
**Taylor (2017)** [88]	**US**	**Quantitative**	**Black, Latinx/Hispanic, White**	**Patient/Caregiver**	**⚫**			
**Sharma (2015)** [89]	**US**	**Quantitative**	**Black, Latinx/Hispanic, White**	**Patient/Caregiver**	**⚫**			
**Campbell (2013)** [90]	**US**	**Quantitative**	**Black, White, Other**	**Patient/Caregiver**	**⚫**			
**Samuel–Ryals (2021)** [91]	**US**	**Quantitative**	**Black, Latinx/Hispanic, White**	**Patient/Caregiver**	**⚫**		⚫	⚫
**Patel (2019)** [92]	**US**	**Quantitative**	**Black, White, Other**	**Patient/Caregiver**				
**Johnson (2020)** [93]	**US**	**Quantitative**	**Black, Latinx/Hispanic, White**	**Patient/Caregiver**	**⚫**			
**Zheng (2011)** [94]	**US**	**Quantitative**	**Black, White**	**Patient/Caregiver**	**⚫**			⚫
**Dosani (2020)** [95]	**Canada**	**Multi–Method**	**South Asian**	**Patient/Caregiver**	**⚫**			
**Zullig (2013)** [96]	**US**	**Quantitative**	**Black, White**	**Patient/Caregiver**	**⚫**			
**Thomas (2013)** [97]	**US**	**Quantitative**	**Black, White**	**Patient/Caregiver**	**⚫**			
**Chung (2016)** [98]	**US**	**Quantitative**	**Black, Latinx/Hispanic, White, Other**	**Patient/Caregiver**	**⚫**			
**Dillon (2016)** [99]	**US**	**Qualitative**	**Black**	**Patient/Caregiver**	**⚫**		⚫	⚫
**Marr (2012)** [100]	**US**	**Quantitative**	**North American Indigenous, Other**	**Patient/Caregiver**	**⚫**			⚫
**Frahm (2012)** [101]	**US**	**Quantitative**	**Black, Latinx/Hispanic, Asian | Multiple Groups, White**	**Patient/Caregiver**	**⚫**			
**Johnson (2019)** [102]	**US**	**Quantitative**	**Black, White, Other**	**Patient/Caregiver**				
**Cole (2019)** [103]	**US**	**Quantitative**	**Black, Latinx/Hispanic, Asian | Multiple Groups, White, Other, Unknown**	**Patient/Caregiver**	**⚫**		⚫	⚫
**Reese (2014)** [104]	**US**	**Qualitative**	**Black**	**Patient/Caregiver**	**⚫**			
**Rizzuto (2018)** [105]	**US**	**Quantitative**	**Black, Latinx/Hispanic, Asian | Multiple Groups, White, Other, Unknown**	**Patient/Caregiver**	**⚫**	⚫	⚫	⚫
**Sharma (2017)** [106]	**US**	**Quantitative**	**Black, White**	**Patient/Caregiver**	**⚫**			
**Fairfield (2012)** [107]	**US**	**Quantitative**	**Black, White, Other**	**Patient/Caregiver**				
**Isaacson (2015)** [108]	**US**	**Qualitative**	**North American Indigenous**	**Provider**	**⚫**			⚫
**Jones (2020)** [109]	**US**	**Quantitative**	**Black, Latinx/Hispanic, Asian | Multiple Groups, White, Other**	**Patient/Caregiver**	**⚫**		⚫	
**Guadagnolo (2014)** [110]	**US**	**Quantitative**	**Asian | Multiple Groups**	**Patient/Caregiver**	**⚫**		⚫	⚫
**Worster (2018)** [111]	**US**	**Quantitative**	**Black, Latinx/Hispanic, White, Unknown, North American Indigenous, Asian/Pacific Islander**	**Patient/Caregiver**	**⚫**			
**Nguyen (2020)** [112]	**US**	**Quantitative**	**Black, Latinx/Hispanic, White, Other**	**Patient/Caregiver**				⚫
**Hardy (2011)** [113]	**US**	**Quantitative**	**Black, Latinx/Hispanic, White, Asian/Pacific Islander**	**Patient/Caregiver**	**⚫**		⚫	⚫
**Kirkendall (2014)** [114]	**US**	**Quantitative**	**Black, Latinx/Hispanic, White, Other, Unknown, Asian/Pacific Islander**	**Patient/Caregiver**	**⚫**			⚫
**DiLuca (2020)** [115]	**US**	**Quantitative**	**Black, Latinx/Hispanic, White**	**Patient/Caregiver**				
**Salomon (2018)** [116]	**US**	**Quantitative**	**Black, Latinx/Hispanic, White**	**Patient/Caregiver**	**⚫**			
**Bajwah (2021)** [117]	**US**	**Quantitative**	**Black, Asian | Multiple Groups, Mixed, White, Other**	**Patient/Caregiver**				
**Check (2016)** [118]	**US**	**Quantitative**	**Black, White**	**Patient/Caregiver**	**⚫**			
**Odejide (2016)** [119]	**US**	**Quantitative**	**Latinx/Hispanic, White, Other**	**Patient/Caregiver**				
**Noh (2015)** [120]	**US**	**Qualitative**	**Black**	**Patient/Caregiver**	**⚫**			⚫
**Russell (2020)** [121]	**US**	**Quantitative**	**Black, Latinx/Hispanic, White, Other**	**Patient/Caregiver**	**⚫**		⚫	⚫
**Abbas (2021)** [122]	**US**	**Quantitative**	**White, Other**	**Patient/Caregiver**	**⚫**			
**Russell (2019)** [123]	**US**	**Multi–Method**	**Black, Latinx/Hispanic, White, Other**	**Patient/Caregiver**				⚫
**Chidiac (2020)** [124]	**UK**	**Quantitative**	**Black, Asian | Multiple Groups, White, Other, Mixed**	**Patient/Caregiver**				
**Kutney–Lee (2017)** [125]	**US**	**Quantitative**	**Black, Latinx/Hispanic, White, Other**	**Patient/Caregiver**	**⚫**			⚫
**Chatterjee (2020)** [126]	**US**	**Quantitative**	**Black, Latinx/Hispanic, White**	**Patient/Caregiver**	**⚫**			
**Chong (2017)** [127]	**US**	**Quantitative**	**Black, Latinx/Hispanic, White, Other**	**Patient/Caregiver**				
**Singh (2017)** [128]	**US**	**Quantitative**	**Black, Latinx/Hispanic, White, Asian/Pacific Islander, North American Indigenous, Unknown**	**Patient/Caregiver**				
**Burgio (2016)** [129]	**US**	**Quantitative**	**Black, White**	**Patient/Caregiver**	**⚫**			
**Lepore (2011)** [130]	**US**	**Quantitative**	**Black, White**	**Patient/Caregiver**	**⚫**			
**Du (2015)** [131]	**US**	**Quantitative**	**Black, White, Other**	**Patient/Caregiver**	**⚫**			
**Mullins (2021)** [132]	**US**	**Quantitative**	**Black, Latinx/Hispanic, White, Other**	**Patient/Caregiver**	**⚫**			⚫
**Thienprayoon (2013)** [133]	**US**	**Quantitative**	**Latinx/Hispanic, White, Other**	**Patient/Caregiver**	**⚫**			
**Frey (2013)** [134]	**New Zealand**	**Qualitative**	**Asian | Multiple Groups, Māori, Asian/Pacific Islander**	**Patient/Caregiver, Provider**	**⚫**			
**D’Angelo (2020)** [135]	**Italy**	**Quantitative**	**Asian | Multiple Groups, White, Black**	**Patient/Caregiver**				⚫
**Nayar (2014)** [136]	**US**	**Quantitative**	**White, Other**	**Patient/Caregiver**	**⚫**			
**Fiala (2020)** [137]	**US**	**Quantitative**	**White, Other**	**Patient/Caregiver**				
**Lee (2021)** [138]	**US**	**Quantitative**	**Black, Latinx/Hispanic, White, Asian/Pacific Islander, Other**	**Patient/Caregiver**	**⚫**			
**Anand (2020)** [139]	**US**	**Quantitative**	**Black, White, Other**	**Patient/Caregiver**				
**Khan (2021)** [140]	**US**	**Quantitative**	**Black, Latinx/Hispanic, White, Asian/Pacific Islander, North American Indigenous, Other**	**Patient/Caregiver**	**⚫**			⚫
**Nuñez (2017)** [141]	**US**	**Qualitative**	**Latinx/Hispanic**	**Patient/Caregiver**	**⚫**			⚫
**Jarosek (2016)** [142]	**US**	**Quantitative**	**Black, Latinx/Hispanic, White, Asian/Pacific Islander**	**Patient/Caregiver**				
**Cote–Arsenault (2019)** [143]	**US**	**Multi–Method**	**Black, Southeast Asian, White, North American Indigenous, Other, Asian/Pacific Islander**	**Patient/Caregiver**	**⚫**			
**Watanabe–Galloway (2014)** [144]	**US**	**Quantitative**	**White, Other**	**Patient/Caregiver**				⚫
**Xian (2014)** [145]	**US**	**Quantitative**	**White, Black, Latinx/Hispanic, Asian | Multiple Groups**	**Patient/Caregiver**	**⚫**			
**Shanmugasundaram (2015)** [146]	**Australia**	**Qualitative**	**South Asian**	**Patient/Caregiver**	**⚫**			⚫
**Sammon (2015)** [147]	**US**	**Quantitative**	**Black, White, Latinx/Hispanic, Other**	**Patient/Caregiver**				
**Fosler (2015)** [148]	**US**	**Quantitative**	**Asian | Multiple Groups, Black, Latinx/Hispanic, White**	**Patient/Caregiver**	**⚫**			
**Allsop (2018)** [149]	**UK**	**Quantitative**	**White, Asian | Multiple Groups, Black, Other, Unknown**	**Patient/Caregiver**				
**Shahid (2013)** [150]	**Australia**	**Qualitative**	**Aboriginal Australian**	**Patient/Caregiver**	**⚫**			⚫
**Saito (2011)** [151]	**US**	**Quantitative**	**White, Other**	**Patient/Caregiver**				
**Tramontano, (2018)** [152]	**US**	**Quantitative**	**White, Black, Latinx/Hispanic, Other**	**Patient/Caregiver**				
**Temkin–Greener (2022)** [153]	**US**	**Quantitative**	**White, Black, Latinx/Hispanic**	**Provider**	**⚫**			
**Kawai (2021)** [154]	**US**	**Quantitative**	**White, Latinx/Hispanic**	**Patient/Caregiver**	**⚫**			
**Soltoff (2022)** [155]	**US**	**Qualitative**	**North American Indigenous**	**Patient/Caregiver**	**⚫**	⚫	⚫	
**Starr, (2022)** [156]	**US**	**Quantitative**	**Asian | Multiple Groups, Black, Latinx/Hispanic, White, Other**	**Patient/Caregiver**	**⚫**			
**Nwogu–Onyemkpa (2022)** [157]	**US**	**Quantitative**	**White, Black, Latinx/Hispanic, Other, Unknown**	**Patient/Caregiver**				
**Price (2021)** [158]	**US**	**Quantitative**	**White, Black, Asian | Multiple Groups, North American Indigenous, Mixed, Other, Unknown, Asian/Pacific Islander**	**Patient/Caregiver**				⚫
**McKee (2022)** [159]	**US**	**Quantitative**	**White, Black, Latinx/Hispanic, Other**	**Patient/Caregiver**	**⚫**	⚫	⚫	⚫
**Beltran (2022)** [160]	**US**	**Qualitative**	**Latinx/Hispanic**	**Patient/Caregiver**	**⚫**			⚫
**Goertz (2022)** [161]	**US**	**Quantitative**	**Black, White, Latinx/Hispanic, Other**	**Patient/Caregiver**				
**Williamson (2022)** [162]	**US**	**Quantitative**	**White, Black, Latinx/Hispanic, Other**	**Patient/Caregiver**	**⚫**			⚫
**Funnell (2021)** [163]	**Canada**	**Quantitative**	**North American Indigenous**	**Patient/Caregiver**	**⚫**			
**DeGroote (2022)** [164]	**US**	**Quantitative**	**White, Black, Asian | Multiple Groups, Latinx/Hispanic**	**Patient/Caregiver**	**⚫**			⚫
**Umaretiya (2021)** [165]	**US**	**Quantitative**	**White, Other**	**Patient/Caregiver**	**⚫**	⚫	⚫	⚫
**Lin, (2022)** [166]	**US**	**Quantitative**	**Black, White, Latinx/Hispanic**	**Patient/Caregiver**	**⚫**	⚫	⚫	⚫
**Zametkin, (2022)** [167]	**US**	**Quantitative**	**White, Black**	**Patient/Caregiver**	**⚫**			
**Bajwah (2021)** [168]	**UK**	**Multi–Method**	**Other**	**Patient/Caregiver**	**⚫**			
**Bandini (2022)** [169]	**US**	**Multi–Method**	**Latinx/Hispanic, White, Black, Asian | Multiple Groups**	**Patient/Caregiver**	**⚫**		⚫	⚫
**Isenberg (2022)** [170]	**Canada**	**Quantitative**	**White, Asian | Multiple Groups, Black, Other, Unknown**	**Patient/Caregiver**			⚫	⚫
**Hunt (2022)** [171]	**US**	**Quantitative**	**White, Black, Latinx/Hispanic, Asian/Pacific Islander, North American Indigenous, Other**	**Patient/Caregiver**				⚫
**Tobin (2022)** [172]	**US**	**Quantitative**	**White, Black**	**Patient/Caregiver**	**⚫**			
**Kiker (2022)** [173]	**US**	**Quantitative**	**White, Black, Asian | Multiple Groups, Other, Latinx/Hispanic**	**Patient/Caregiver**	**⚫**	⚫	⚫	⚫
**Mahilall (2021)** [174]	**South Africa**	**Qualitative**	**Other, White, Black, Mixed**	**Provider**	**⚫**		⚫	⚫
**Shepard (2022)** [175]	**US**	**Quantitative**	**Latinx/Hispanic**	**Patient/Caregiver**	**⚫**			⚫
**Turkman (2019)** [176]	**US**	**Quantitative**	**White, Other**	**Patient/Caregiver**				
**Cicolello (2019)** [177]	**US**	**Qualitative**	**Black, White, Other, Latinx/Hispanic, Asian | Multiple Groups**	**Patient/Caregiver, Provider**	**⚫**		⚫	⚫
**Smith (2018)** [178]	**US**	**Quantitative**	**White, Black, Latinx/Hispanic, Other**	**Patient/Caregiver**				
**Noh (2022)** [179]	**US**	**Quantitative**	**White, Other**	**Patient/Caregiver**	**⚫**			
**Estrada (2022)** [180]	**US**	**Quantitative**	**Black, Latinx/Hispanic**	**Patient/Caregiver**	**⚫**		⚫	⚫
**Giap (2023)** [181]	**US**	**Quantitative**	**White, Black, Asian/Pacific Islander**	**Patient/Caregiver**	**⚫**			⚫
**Soipe (2023)** [182]	**US**	**Quantitative**	**White, Black, Hispanic/LatinX, Other, Turtle Island (North American) Indigenous, Asian | Multiple Groups, Asian/Pacific Islander, Unknown**	**Patient/Caregiver**				
**Khayal (2023)** [183]	**US**	**Quantitative**	**White, Other**	**Patient/Caregiver**	**⚫**	⚫	⚫	⚫
**Fisher (2023)** [184]	**US**	**Quantitative**	**White, Black, Hispanic/LatinX, Other**	**Patient/Caregiver**	**⚫**			
**Ali (2022)** [185]	**US**	**Quantitative**	**White, Black, Hispanic/LatinX, Other, Asian/Pacific Islander, Turtle Island (North American) Indigenous**	**Patient/Caregiver**	**⚫**			
**Jin (2022)** [186]	**US**	**Quantitative**	**White, Black, Hispanic/LatinX, Asian | Multiple Groups**	**Patient/Caregiver**	**⚫**		⚫	⚫
**Anderson (2022)** [187]	**US**	**Qualitative**	**White, Other, Turtle Island (North American) Indigenous**	**Patient/Caregiver**	**⚫**		⚫	⚫
**Hunt (2023)** [188]	**US**	**Quantitative**	**White, Black, Hispanic/LatinX, Asian/Pacific Islander**	**Patient/Caregiver**	**⚫**	⚫	⚫	⚫
**Anandarajah (2023)** [189]	**US**	**Qualitative**	**White, Black, Hispanic/LatinX, Other, Asian | Multiple Groups, Mixed**	**Patient/Caregiver, Provider**	**⚫**			
**Siddiqui (2023)** [190]	**US**	**Quantitative**	**White, Black, Other, Asian | Multiple Groups, Asian/Pacific Islander, Turtle Island (North American) Indigenous, Unknown**	**Patient/Caregiver**	**⚫**		⚫	
**Peeler (2023)** [191]	**US**	**Quantitative**	**White, Black, Hispanic/LatinX, Other, Asian | Multiple Groups**	**Patient/Caregiver**				
**Dewhurst (2023)** [192]	**UK**	**Qualitative**	**Black**	**Patient/Caregiver**	**⚫**	⚫	⚫	⚫
**Elkaryoni (2022)** [193]	**US**	**Quantitative**	**White, Black, Hispanic/LatinX, Other, Asian | Multiple Groups, Turtle Island (North American) Indigenous**	**Patient/Caregiver**				
**Aamodt (2023)** [194]	**US**	**Quantitative**	**White, Black, Hispanic/LatinX, Other, Asian | Multiple Groups, Turtle Island (North American) Indigenous**	**Patient/Caregiver**	**⚫**	⚫	⚫	
**Weerasinghe (2023)** [195]	**Canada**	**Qualitative**	**South Asian**	**Patient/Caregiver**	**⚫**			⚫
**Fassas (2023)** [196]	**US**	**Quantitative**	**White, Black, Hispanic/LatinX, Other, Asian | Multiple Groups**	**Patient/Caregiver**	**⚫**	⚫	⚫	
**Zapata (2023)** [197]	**US**	**Quantitative**	**White, Black, Hispanic/LatinX, Asian | Multiple Groups, Asian/Pacific Islander, Turtle Island (North American) Indigenous, Unknown**	**Patient/Caregiver, Provider**	**⚫**			⚫
**Tabuyo-Martin (2023)** [198]	**US**	**Quantitative**	**White, Black, Hispanic/LatinX, Asian | Multiple Groups, Unknown**	**Patient/Caregiver**	**⚫**			
**Moriyama (2021)** [199]	**US**	**Quantitative**	**White, Black, Hispanic/LatinX, Other, Unknown**	**Patient/Caregiver**				⚫
**Johnson (2012)** [200]	**US**	**Quantitative**	**White, Black**	**Patient/Caregiver**				
**Penn (2014)** [201]	**US**	**Quantitative**	**White, Black, Hispanic/LatinX, Other, Asian/Pacific Islander**	**Patient/Caregiver**	**⚫**			⚫
**Holland (2015)** [202]	**US**	**Quantitative**	**White, Black, Other, Asian/Pacific Islander, Turtle Island (North American) Indigenous, Unknown**	**Patient/Caregiver**				
**Accordino (2017)** [203]	**US**	**Quantitative**	**White, Black, Other**	**Patient/Caregiver**				
**Alqahtani (2019)** [204]	**US**	**Quantitative**	**White, Black, Hispanic/LatinX**	**Patient/Caregiver**				
**Elting (2020)** [205]	**US**	**Quantitative**	**White, Black, Hispanic/LatinX, Other**	**Patient/Caregiver**	**⚫**			
**Leibowitz (2020)** [206]	**US**	**Quantitative**	**White, Black, Hispanic/LatinX, Other**	**Patient/Caregiver**				
**Wasp (2020)** [207]	**US**	**Quantitative**	**White, Other**	**Patient/Caregiver**	**⚫**			⚫
**Chu (2021)** [208]	**US**	**Quantitative**	**White, Black, Hispanic/LatinX, Other, Asian/Pacific Islander, Turtle Island (North American) Indigenous**	**Patient/Caregiver**		⚫		

^a^A black circle indicates a yes value for the column (e.g., Race/ethnicity was a major focus of the research purpose, the authors named racism in the discussion, etc.)

^b^Keywords include bias, discriminat*, systemic, prejudic*, or stereotype

As seen in Table 5, almost 90% of studies were conducted in the United States of America (US) followed by Australia and Canada, each contributing 3.1% of studies. The top four categories of race or ethnicity utilized were White (81.22% of studies), followed by Black (73.48%), Latinx/Hispanic (53.59%), and Other (53.04%). Nearly two thirds (74.03%) of studies included four or fewer categories for race or ethnicity, and 93.48% focused on the race or ethnicity of the patient or caregiver.

**Table 5:** Summary of study characteristics

Characteristic	*n*	%
**Total number of citations**	181	--
**Annual rate** median(range)	12 (8–24)	--
**Study Country**		
United States of America	161	88.95%
Australia	6	3.31%
Canada	6	3.31%
UK	4	2.21%
New Zealand	2	1.10%
South Africa	1	0.55%
Italy	1	0.55%
**Study Design**		
Quantitative	150	82.87%
Qualitative	26	14.36%
Multi-method	5	2.76%
**# Races/Ethnicities Investigated**		
1	30	16.57%
2	39	21.55%
3	26	14.36%
4	39	21.55%
5	26	14.36%
> 5	21	11.60%
**Race/Ethnicities included**^**a**^		
White	147	81.22%
Black	133	73.48%
Hispanic/LatinX	97	53.59%
Other	96	53.04%
Asian | Multiple	46	25.41%
Asian/Pacific Islander	26	14.36%
Turtle Island (North American) Indigenous	24	13.26%
Unknown	17	9.39%
Aboriginal Australian	5	2.76%
Mixed	6	3.31%
Māori	2	1.10%
South Asian	3	1.66%
East Asian	2	1.10%
Southeast Asian	1	0.55%
**Race/Ethnicity presented for**		
Patient or Caregiver	169	93.37%
Healthcare provider	6	3.31%
Both	6	3.31%
**Focus of race/ethnicity to the research purpose**		
Major Focus	129	71.27%
Minor Focus	52	28.73%
**Authors named racism in the discussion**		
Yes	13	7.18%
No	168	92.82
**Authors used a keyword in the discussion**^**b**^		
Yes	37	20.44%
No	144	79.56%
**Authors described systemic or individual provider factors in the discussion**		
Yes	75	41.44%
No	106	58.56%

^a^Select all that apply; the overall count will amount to more than 181

^b^Keywords are bias, discriminat*, systemic, prejudic*, or stereotype

**Fig. 2:**
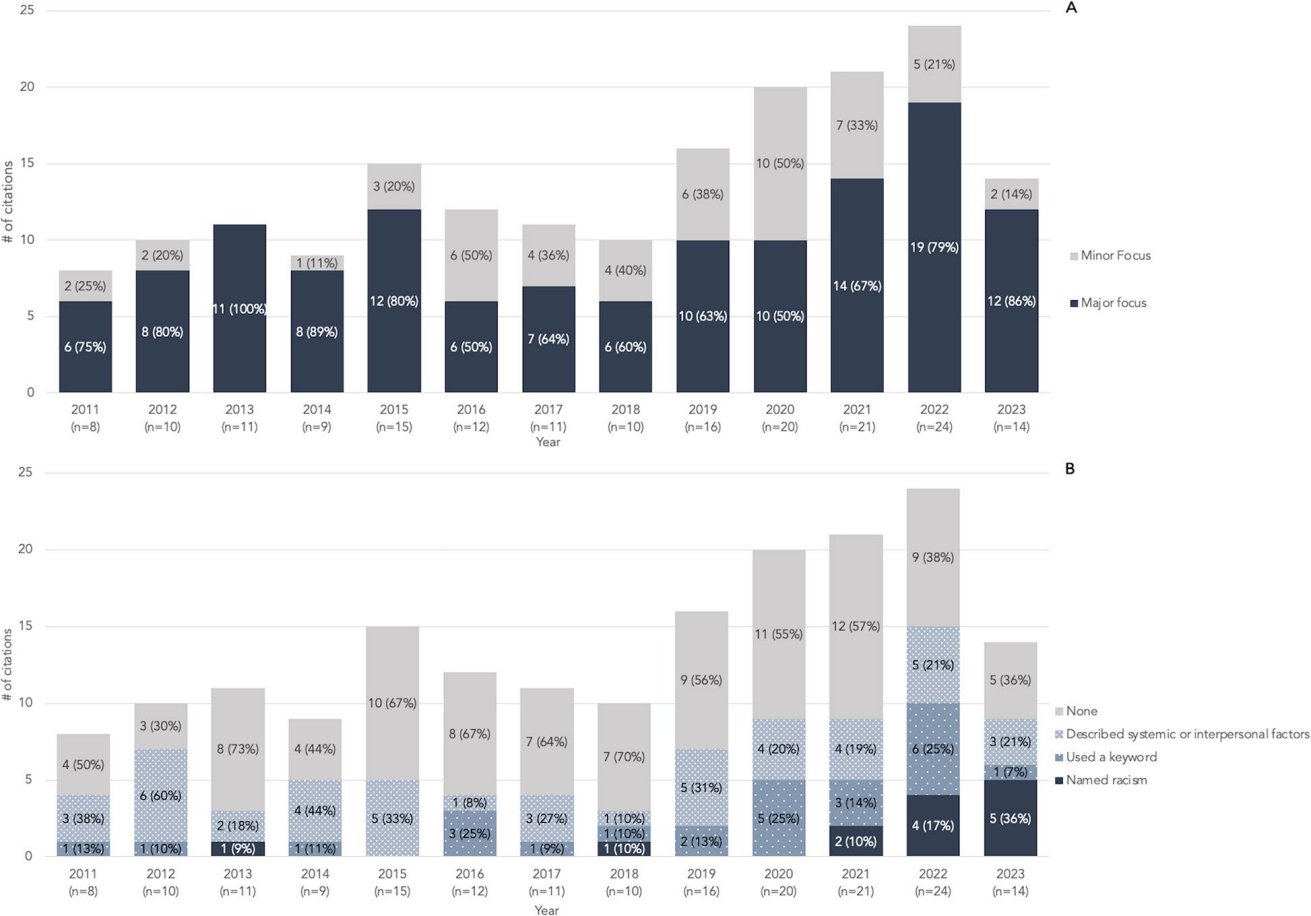
Research focus and interpretation of findings by year.  **A** Number of citations where race or ethnicity were the major focus or minor focus of the stated research purpose by year. **B** Number of citations that named racism, used a keyword, described systemic or individual provider factors, or none by year. Keywords include ‘bias’, ‘discriminat*’, ‘systemic’, ‘prejudic*’, and ‘stereotype’. Percent is calculated using the number of citations for that year as the denominator. 2023 includes up to October 17, 2023, only

Race or ethnicity were a major focus of most studies (71.27%). The proportion of studies with major focus on race or ethnicity fluctuated from year to year with a low of 50% of articles in 2016, and a high of 100% of articles in 2013 (Fig. 2A). Within the discussion sections, authors described systemic or individual provider factors (41.44%) more often than they used a keyword (20.44%). Only a small portion (7.18%) directly named racism. The number of studies that discussed racism, either directly or indirectly, steadily increased since 2018, although the total number annually remains low (Fig. 2B): In 2013, 27% of articles discussed racism and this has gradually increased to 64% of articles in 2023. Since 2021, authors of 11 out of 33 articles (33.33%) directly named racism when interpreting findings representing an increase from the previous ten years (2011–2020), in which only two out of 51 articles (3.92%) directly named racism.

### Intersection between research focus and interpretation of findings

**Fig. 3:**
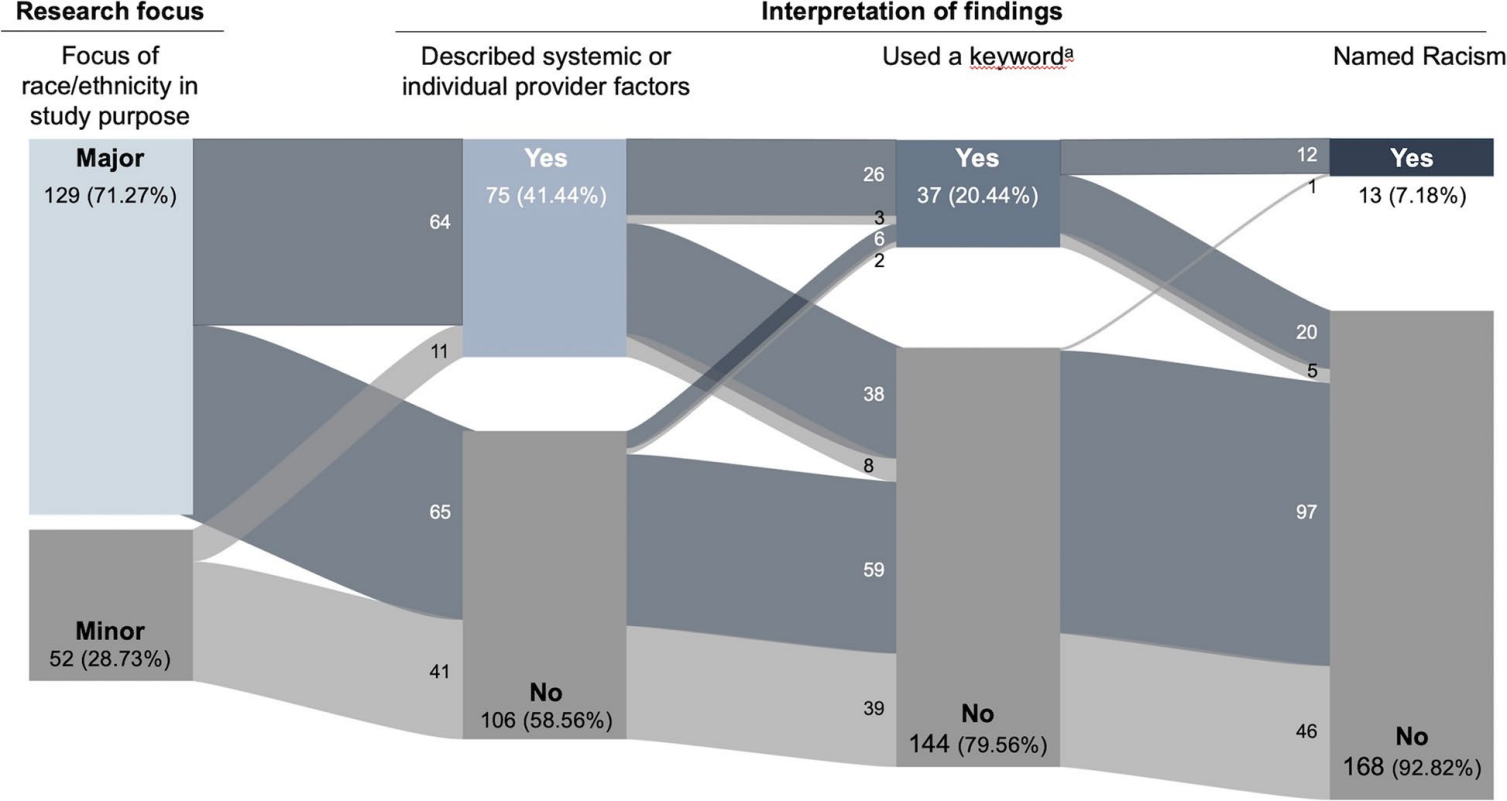
Sankey diagram of citations by research focus and interpretation of findings.  This sankey diagram shows the flow of citations from research focus (whether race or ethnicity were a major or minor focus of the stated research objective) across interpretation of findings, from indirect to direct. The colour of the path indicates research focus: Blue paths denote citations that had a major focus on race or ethnicity, and grey paths denote citations that had a minor focus on race or ethnicity. Keywords include ‘bias’, ‘discriminat*’, ‘systemic’, ‘prejudic*’, or ‘stereotype’. Percents are calculated using the size of the sample ( *n*  = 181) as the denominator.

Figure 3 shows the flow of studies from their focus on the left across the various forms of acknowledging racism in the interpretation of findings. Articles with a major focus on race or ethnicity were more likely to discuss racism directly or indirectly. Of the 13 studies that directly named racism, 12 (92.31%) included the examination of race or ethnicity as a major focus of their research questions. Similarly, 32 of 37 (86.49%) studies that used a keyword and 64 of 75 (85.33%) studies that described systemic or individual provider factors started with race or ethnicity as a major focus of their research question. If authors directly discussed racism in their findings, it is likely that they also did so indirectly: 12 of 13 (92.31%) articles that named racism also used a keyword, and 29 of 37 (78.38%) articles that used a keyword also described systemic or individual provider factors.

## Discussion

In this study examining if and how palliative care researchers discuss racism when contextualizing their findings, we observed that only 7.18% of studies directly named racism in their discussions despite 71.27% including race or ethnicity as a major focus of their research purpose.

Overall, the paucity of explicit discourse on racism in the palliative care literature we reviewed mirrors the general medical literature where the word “racism” appeared in less than 1% of articles in four of the highest impact general medical journals [209]. Low rates of the word “racism” observed in our findings could have several explanations. Researchers may not have considered systemic or interpersonal racism as causes for the observed outcomes. Although the use of the word racism was low, authors more often described systemic or individual provider factors. It is possible that researchers directly or indirectly discuss racism within their interpretations, but were censored or encouraged to ‘soften their language’ by medical journal reviewers or editors in order to make arguments more palatable to scientific audiences [210].

The time trends visualizing the proportion of articles that directly or indirectly discuss racism exhibits two peaks in 2012 and 2022–2023 that seem to coincide with societal events and subsequent movements that brought discussions of race and racism to the fore, particularly in the United States and Canada. The murders of Trayvon Martin in 2012 and George Floyd in 2020—Black men in the US—and the confirmation of mass unmarked graves at Canadian residential schools in 2021—government-sponsored schools designed to assimilate Indigenous children who were subjected to neglect, medical experimentation and abuse [211]—each spurred social movements, including Black Lives Matter, and increased societal awareness of inequities and injustices faced by racialized people. As well, many racialized communities had higher case fatality rates during the COVID-19 pandemic, drawing additional attention to existing health and social inequities for racialized people [212–214]. Since 2020, advocacy for racial equality has prompted governments, health care institutions, academic organizations, and medical journals to create new policies and standards [215–217] – including the importance of naming and discussing racism in health disparities research, which may explain the upward and seemingly sustained trend we observed over the past three years.

Examining the flow of citations across research focus and interpretation of findings, we found that authors were more likely to directly or indirectly discuss racism when contextualizing their findings if race or ethnicity were a major focus of a study, and that more direct references to racism were less common than indirect references to racism. This may indicate that researchers with greater understanding of the root causes of inequities for racialized and ethnically minoritized groups are more likely to design research focused on detecting and describing race- and ethnicity-based disparities in palliative care, and more likely to articulate these factors in the interpretation of their findings.

We found little diversity in the representation of studies in both the races or ethnicities that were researched, nor in the geographic location of the research. First, most studies examined outcomes for people who were White, suggesting that this population may have been used as a comparator group. This approach has been criticized for centering Whiteness and placing many diverse groups within the Other [178] and perpetuating a broader system where White race is considered normative, and outcomes for individual racialized groups are obscured [218]. While White, Black, Hispanic/LatinX groups were examined in more than 50% of citations, others –– including Turtle Island (North American) Indigenous, Aboriginal Australian, Asian (multiple groups), Asian/Pacific Islander, Mixed, Māori, South Asian, East Asian, and Southeast Asian – were named in 25% or fewer studies. This lack of representation highlights groups that could potentially benefit from more focus in palliative care research (for example Southeast Asian, East Asian, South Asian, Indigenous groups, West Asian/Middle Eastern, mixed race etc.).

Second, a vast majority of the identified studies originated in the US with little representation from other countries. This finding suggests a significant overrepresentation of US-based studies when compared with published palliative care research, generally. A recent bibliometric review and mapping analysis of international palliative care research reported that the US represented 31.53% of articles, followed by the UK (12.58%), Canada (8.26%) and Australia (6.25%) [219]. This paucity of geographic diversity may indicate differing conceptualizations of race or ethnicity between countries. Previous research suggests that the US may be overrepresented in the area of racial or ethnic disparities research because the concept of race is legally defined and frequently collected, making this data more available to researchers [220]. While the concept of race and ethnicity can vary geographically and there may be challenges with data collection in some jurisdictions, it remains important to pursue research in geographically underrepresented locations. Research is a key foundational component of improving national palliative care systems [221, 222], therefore it is imperative that research from under-represented populations and countries be supported and prioritized. Supports may include increasing funding available for research [222], establishing peer-reviewed publication targets [221], and cultivating national and international congresses or scientific meetings that prioritize palliative care research, facilitate capacity building, and encourage the formation of collaborative relationships between different sectors and countries [219, 221].

### Limitations

First, because race is socially constructed, its definitions, meanings, and racial categories themselves can change depending on the larger context. Researchers will therefore conceptualize race based on their own setting, which may result in some knowledge being missed by our search strategy. Similarly, concepts may have different names in different languages and studies in languages other than English may have not been captured in our search for this reason.

Second, when analyzing study characteristics, we adopted racial and ethnic categories consistent with those used in the included studies. These categories are often problematic as they erroneously group these distinct concepts together and centre on North American academic research norms. While this approach is widely applied in medical research, it often confounds race and ethnicity, which may impact the ability to analyse and discuss the content in a more robust way.

Racism has profound and pervasive effects on many aspects of a person’s health. However, the examination of racism alone does not give a complete picture of a nuanced social experience. Other factors related to one’s identity also contribute to health inequities for racialized and ethnically minoritized individuals. These components are often captured in social determinants of health and intersectionality frameworks and include categories such as sexism, access to education, discrimination related to social class, gender identity, etc [223]. Other important related factors may have been missed by limiting the analysis to racism.

Finally, when examining if authors discussed systemic individual provider factors in the interpretation of their findings, there was no pre-existing list of keywords. Although we created our own list of terms based on existing recommendations and guidelines, this may have resulted in a list that is too specific or too sensitive. For example, if the keyword list we created was too sensitive, it may have included some instances where racism was not necessarily directly named, resulting in an overcounting of the articles categorized as directly naming racism.

### Recommendations

Based on our findings, we propose three groups of recommendations for palliative care researchers:

1. Researcher education

Prior to beginning studies exploring race, ethnicity or racism, researchers should educate themselves about these topics and how they work as underlying mechanisms of health disparities. Evidence informed guides based on consultations from affected communities are available with concrete, detailed advice on how to approach and frame questions of enquiry [223].

2. Research planning

Researchers must design their research questions with intention, grounded in evidence-based understandings of underlying mechanisms, such as systemic racism. They must also ensure that the data sets are collected and constructed to meaningfully meet the research objectives. If partitioning based on race or ethnicity is required for data analysis, it is important to avoid unnecessary groupings that may obscure findings for certain individual groups. If comparisons are warranted, researchers should avoid using White as the normative comparator. Rather, consider the reason and justification for comparison, avoiding automatic comparison to the perceived dominant group. Some alternate approaches include comparisons to the group’s historical data or to the population average [223]. Additionally, researchers should make sure that race and ethnicity are not conflated. If they are used together, this should be justified based on the researchers’ context or available data sets.

3. Interpretation of findings

An evidence-based understanding of underlying root causes of health disparities, including the role of racism and its manifestations, should be used to guide the interpretation of findings. Differences should not be attributed to patient factors without evidence, as this may reflect researchers’ underlying biases and risks perpetuating stereotypes, and worsening disparities [15, 217].

## Conclusion

Although the volume of palliative care literature examining the impacts of race or ethnicity on palliative care outcomes and experiences is increasing, there remains significant room for improvement when it comes to recognizing the role of systemic factors including racism when interpreting findings. Our study found that only a small proportion of researchers who deliberately set out to explore race or ethnicity explicitly named racism as a possible root cause. Researchers hold significant influence over the trajectory of health disparities research through the manner in which they frame, conduct, and interpret their studies. Failing to name evidence-based root causes such as systemic and interpersonal racism — because of lack of knowledge, fear, censoring, or just not considering it — threatens the effectiveness of future studies or interventions to address health disparities. Further, omitting the concept of racism when exploring racial and ethnic disparities may do harm by further reinforcing systems of oppression that affect racialized patients in need of palliative care.

### Supplementary Information

Supplementary Material 1.

Supplementary Material 2.

## Data Availability

No datasets were generated or analysed during the current study.
