# Supplementary material for: Naming racism as a root cause of inequities in palliative care research: a scoping review
Source: BMC Palliat Care. 2024 Jun 10;23:143. doi: 10.1186/s12904-024-01465-9 (PMC11163751; doi:10.1186/s12904-024-01465-9)
Supplement: Supplementary file 1 — Supplementary Material 1. [file 12904_2024_1465_MOESM1_ESM.docx]

Additional File 1: Search Strategy

*A comprehensive list of databases and search terms used to search the literature*

Databases:

- CINAHL
- Cochrane
- EMBASE
- MEDLINE
- PubMed

Database: Ovid MEDLINE(R) ALL <1946 to July 06, 2021> Search Strategy:

--------------------------------------------------------------------------------

1     continental population groups/ or african continental ancestry group/ or african americans/ or american native continental ancestry group/ or indians, central american/ or indians, north american/ or alaskan natives/ or indigenous canadians/ or inuits/ or american natives/ or indians, south american/ or asian continental ancestry group/ or asian americans/ or european continental ancestry group/ or oceanic ancestry group/ (230701)

2     ethnic groups/ or indigenous peoples/ or inuits/ (161826)

3     Palliative Care/ (57163)

4     "Hospice and Palliative Care Nursing"/ (1317)

5     Hospices/ (5223)

6     Terminal Care/ (29532)

7     Hospice Care/ (6937)

8     Suicide, Assisted/ (5822)

9     Refusal to Treat/ (3054)

10     Racism/ (3667)

11     racism.ti. (1812)

12     ((racial or ethnic*) adj1 (bias or discrimin* or prejudic* or disparit* or equit* or inequit* or equality or inequality*)).ti. (5487)

13     ethnicity.ti. (8120)

14     palliative.ti. (26638)

15     "terminal care".ti. (770)

16     "end of life".ti. (11692)

17     hospice*.ti. (6794)

18     "refusal to treat".ti. (29)

19     race.ti. (16514)

20     3 or 4 or 5 or 6 or 7 or 8 or 14 or 15 or 16 or 17 (97163)

21     9 or 10 or 11 or 12 (12774)

22     20 and 21 (242)

23     13 or 18 or 19 or 21 (33708)

24     20 and 23 (341)

25     1 or 2 or 23 (323814)

26     20 and 25 (1325)

27     24 not 26 (0)

28     26 not 24 (984)

29     limit 26 to yr="2011 -Current" (673)
