# Supplementary material for: Naming racism as a root cause of inequities in palliative care research: a scoping review
Source: BMC Palliat Care. 2024 Jun 10;23:143. doi: 10.1186/s12904-024-01465-9 (PMC11163751; doi:10.1186/s12904-024-01465-9)
Supplement: Supplementary file 2 — Supplementary Material 2. [file 12904_2024_1465_MOESM2_ESM.docx]

# Additional File 3: List of data abstraction variables

*A list of variables that were collected from each citation*

| Citation Data | 1. First author 2. Title 3. Publication year 4. Location of study (Select all that apply)    1. Canada    2. USA    3. UK    4. Other (Free text) 5. Study purpose (Free text) 6. Study outcomes (Free text) |
| --- | --- |
| Races and/or Ethnicities collected | (Select all that apply)   1. Black (Including African, Caribbean, etc.) 2. White 3. LatinX/Hispanic 4. Asian (Multiple) 5. South Asian (Afghanistan, Bangladesh, Bhutan, India, Nepal, Pakistan, Sri Lanka, Maldives) 6. East Asian (China, Japan, Mongolia, North Korea, South Korea, Taiwan) 7. Southeast Asian (Brunei, Cambodia, East Timor, Indonesia, Laos, Malaysia, Myanmar, Philippines, Singapore, Thailand, Vietnam) 8. Other (if a named race or ethnicity, type in the name. If the study specifies other, type “other”). 9. Unknown/Not specified |
| Whose race was collected? | (Select all that apply)   1. Patient/caregiver 2. Provider |
| Research Intention  (Was race/ethnicity identified as a primary focus in the study purpose) | (Select one)   1. Yes 2. No |
| Research phase | (Select one)   1. Detect 2. Understand 3. Both |
| Interpretation of findings  (Did the study authors explicitly or non-explicitly identify systemic factors as a potential explanation for differences seen between racial or ethnic populations?) | 1. Did they use a keyword? (Select one)    1. Yes    2. No 2. Was racis* specifically used? (Select one)    1. Yes    2. No 3. Did they allude to system level factors? (Select one)    1. Yes    2. No |
